# A Repair Method for Damage in Aluminum Alloy Structures with the Cold Spray Process

**DOI:** 10.3390/ma14226957

**Published:** 2021-11-17

**Authors:** Xiaohui Han, Xiaoguang Sun, Gangqing Li, Shiming Huang, Ping Zhu, Chunyuan Shi, Tengfei Zhang

**Affiliations:** 1 CRRC Qingdao Sifang Co., Ltd., Qingdao 266111, China; hanxiaohui@cqsf.com (X.H.); sunx_sf@126.com (X.S.); ligangqing@cqsf.com (G.L.); 2 School of Materials Science and Engineering, Dalian Jiaotong University, Dalian 116028, China; zhuping@djtu.edu.cn (P.Z.); shicy@sina.com (C.S.); ztf19981207@163.com (T.Z.)

**Keywords:** cold spray process, corrosion resistance, mechanical properties, 7N01

## Abstract

Aluminum alloy structures may be damaged due to wear or corrosion while in service. These damages will bring about huge financial costs, as well as a huge amount of energy consumption. There is an urgent need to search for an appropriate repair method in order to solve this problem. In this research, the cold spray process was used to repair the damages by using a mixture of powders with Al and Al_2_O_3_. A 7N01-T4 aluminum alloy plate with a factitious pit was regarded as the damaged sample. The microstructure, mechanical properties, and corrosion behavior were studied. The results showed that there were no visible perforative pores or cracks in the repaired areas. The microhardness of the repaired areas was in the range of 57.4–63.2 HV and was lower than that of the 7N01-T4 aluminum alloy. The tensile strength of the repaired samples was markedly improved compared with the unrepaired samples. The alternate immersion test results indicated that the repaired samples had the lowest rate of mass loss compared with 7N01-T4 and the unrepaired samples. After alternate immersion tests for 504 h, the repaired samples were covered with dense corrosion products. The repaired samples had a superior corrosion resistance compared to that of 7N01-T4. Thus, the cold spray process is a method of repairing damage in aluminum alloy structures.

## 1. Introduction

Aluminum alloys have been applied in the manufacturing industry due to their excellent performance in corrosion resistance, specific strength, and workability. However, aluminum alloy structures may be damaged due to wear or corrosion when they are in service in harsh and complex environments. For example, the car bodies of high-speed trains made from aluminum alloys may suffer from corrosion and aging in complex atmospheric environments [1,2,3]. In addition, the car bodies may be partially injured under the impact of external forces, such as scratches, pits, and laceration. The damages caused by these factors bring about a large energy consumption and weaken the durability and safety of high-speed trains that are in service. Thus, it is urgent to propose a perfect method of repairing such damages in order to solve these problems.

The cold spray process is a new surface-repairing method with many advantages, such as a low-heat effect on the substrate, a dense microstructure, and excellent mechanical properties [4,5,6,7,8,9,10]. Zhang et al. [11] prepared Al-based coatings on the AA 2024-T3 alloy. The coatings were dense, and no perforative pores or cracks were observed. Due to the impact of the particles at high speed, there was a compressive stress in the coatings. This was beneficial for improving the mechanical properties and wear resistance. The Al 5083 coatings showed better mechanical and anti-corrosion properties. Cong et al. [12] used Al-Al_2_O_3_ powders to prepare composite coatings on steel and studied the wear behavior. After being exposed in 5 wt.% NaCl solution for 960 h, the coatings still had competent protection for the substrate. However, the wear resistance of the corroded coatings was decreased because of the accumulation of damage. Shockley et al. [13] used in-situ tribometry to study the wear behavior of Al-Al_2_O_3_ coatings prepared with the cold spray process. They found that the existence of Al_2_O_3_ particles enhanced the stability of coatings during the wear process. In addition, Al-Al_2_O_3_ coatings were also successfully deposited by using the cold spray process on a magnesium alloy substrate [14,15], steel substrate [16], and polymer matrix composite [17]. Hence, cold-sprayed coatings can improve the mechanical properties and corrosion resistance. There are many considerations when choosing a preparation process for damage in a car body. It was mentioned that there are many electrical systems around the damaged positions in a car body, so these areas catch fire easily. Some technologies, such as metal inert gas welding, are not applicable. Moreover, the thermal spray process is likewise limited because thermal deformation will easily occur. As a result, the difficulty and the workload will be increased in the subsequent processing. Therefore, the cold spray process is an ideal method for repairing the damaged components of the car bodies of high-speed trains [18].

In this research, the cold spray process was used to prepare a common material for a car body of a high-speed train. The microstructure, mechanical properties, and corrosion behavior of the repaired samples were studied. A comparative study with unrepaired samples was also conducted. This research will provide essential data and have great significance for the safety and the stability of high-speed trains.

## 2. Materials and Methods

### 2.1. Materials

**Figure 1:**
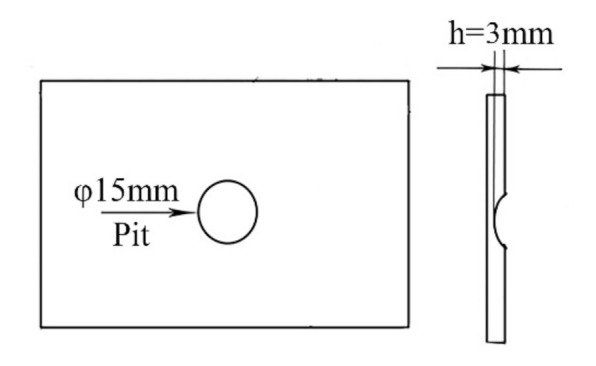
**A** schematic diagram of the samples with a factitious pit.

The 7N01-T4 aluminum alloy, which is used as a common material for high-speed trains, was chosen as the substrate. The chemical compositions are listed in Table 1. For the purpose of simulating the damages produced in high-speed trains while in motion, factitious pits with a thickness of 3 mm were produced in the 7N01-T4 plates in advance. Figure 1 shows a schematic diagram of the damaged samples.

**Figure 2:**
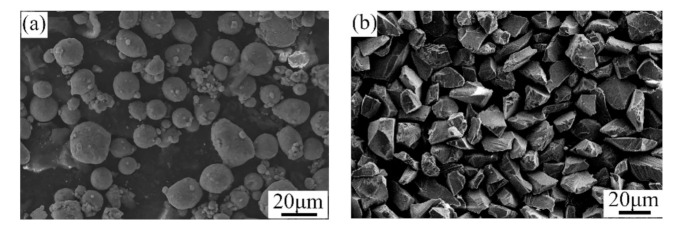
SEM morphologies of the (**a**) Al and (**b**) Al_2_O_3_ powders.

A mixture of pure Al and Al_2_O_3_ powders was utilized to repair the factitious damages with the cold spray process. The mass ratio of the pure Al and Al_2_O_3_ powders was 76 to 24. All of the powders were produced by the Tyontech Company Limited in Xi’an in China. Figure 2 shows the SEM morphologies of the Al and Al_2_O_3_ powders. As depicted, the particles of the pure Al powder were spherical, and the particle size was in the range of 10–30 μm. The particles of the Al_2_O_3_ powder were angular and had an average size of 20–40 μm.

### 2.2. Methods

**Figure 3:**
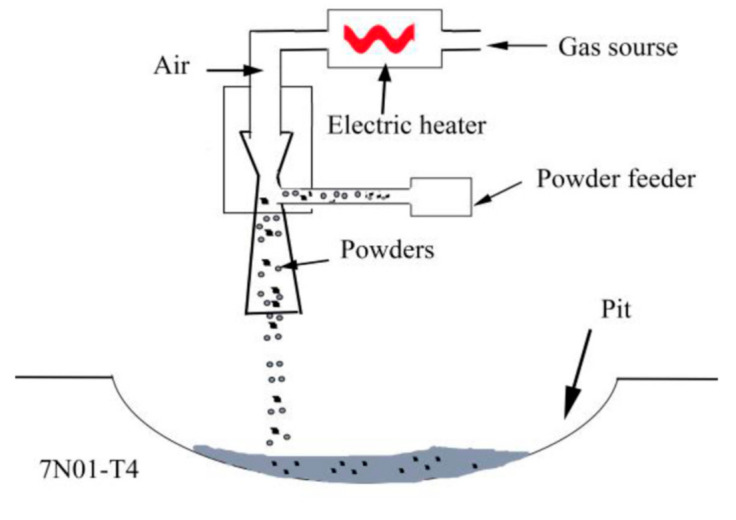
A schematic illustration of the repair process.

Before being repaired, the aluminum alloy plates were fully ground with an angle grinder to remove the surface oxidation film, and were then wiped with acetone. The mixed powders were kept in a holding furnace at 60 °C for 3 h. The samples were repaired with a low-pressure cold spray system (Tyontech Co., LTd, Xi’an, China). A schematic illustration of the repair process is shown in Figure 3. The process parameters were as follows: process gas, air; pressure, 0.6 MPa; preheating temperature, 350 °C; powder feed rate, 25 g/min; gas flow rate, 0.4 L/min. After the repair, the excess parts in the repaired areas were removed to ensure that the surface was absolutely flat. Then, the repaired samples were processed into different sizes for microstructural analyses, mechanical property tests, and corrosion tests.

The samples for the microstructural analysis were ground with sandpapers and polished with polishing agents. A mixed solution with 95 mL of H_2_O, 2.5 mL of HNO_3_, 1.5 mL of HCl, and 1.0 mL of HF was used to etch the samples. The microstructure was studied with an optical microscope (OM, OLYMPUS, Tokyo, Japan) and a field-emission scanning electron microscope (SEM, SUPRA55, ZEISS, Jena, Germany).

The Vickers hardness on the transverse sections of the samples was assessed with a Vickers hardness tester (FM-300, FUTURE TECH, Tokyo, Japan) with a test load of 4.9 N for 15 s. A universal tester (WDW-300E, Jinan New Gold Test Instrument Co., LTd, Jinan, China) was used for the transverse tensile tests according to EN ISO 4136–2012. Three samples were used as a group to obtain the average tensile strength.

To study the corrosion behavior, alternate immersion tests in a salt solution were conducted according to ISO 11130–2017 [19]. The salt solution was 50 g/L of NaCl solution, which was mixed with NaCl and deionized water. Then, the pH was adjusted to 3.5 through the addition of HNO_3_, H_2_SO_4_, and NaOH solution. One test cycle involved immersion in the solution at 25 °C for 10 min and drying at 70 °C for 50 min. Before and after the tests, the samples were weighed with an analytical balance. Four samples were used as a group to obtain the average rate of mass loss. The rate of mass loss was calculated with the following formula:

*R* = (*W_b_* − *W_a_*)/S, (1)

R—the rate of mass loss,

*W_b_*—the mass of samples before tests,

*W_a_*—the mass of samples after tests,

*S*—the superficial area for tests.

The electrochemical tests on the samples were conducted on an electrochemical workstation (CHI 700E, Chinstruments Co., LTd, Shanghai, China) according to ISO 17475–2005 [20]. Three electrodes were used: a working electrode (the samples with an area of 1 cm^2^), reference electrode (saturated calomel electrode), and auxiliary electrode (platinum sheet). The test solution was a 3.5% NaCl solution. The open circuit potential (*E*_OCP_) of the samples before the alternate immersion tests was tested for 0.5 h. After the alternate immersion, the potentiodynamic polarization was tested with a scanning rate of 0.33 mV/s from −0.5 to 1.5 V versus the OCP.

## 3. Results and Discussion

### 3.1. Microstructure

**Figure 4:**
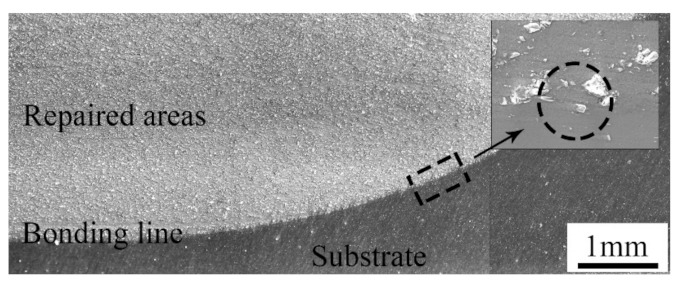
Cross-section of samples repaired with the cold spray process.

The cross-section of the samples repaired with the cold spray process is shown in Figure 4. As can be seen, the repaired areas were relatively dense. There were no visible pores and cracks that penetrated the entire cross-section. Thus, it was not easy for a corrosive medium to directly infiltrate into the inner substrate [21,22]. In addition, a boundary between the repaired areas and the substrate was apparent. The bonding line was along the surface of the pit, that is to say, no metal in the substrate was melted during the cold spray process. The bonding line was not always smooth, and some hollow areas were obvious in some positions. As shown in the magnified image, many angular particles were embedded in the substrate. This illustrates that a good mechanical bond between the repaired areas and the substrate was formed [23].

**Figure 5:**
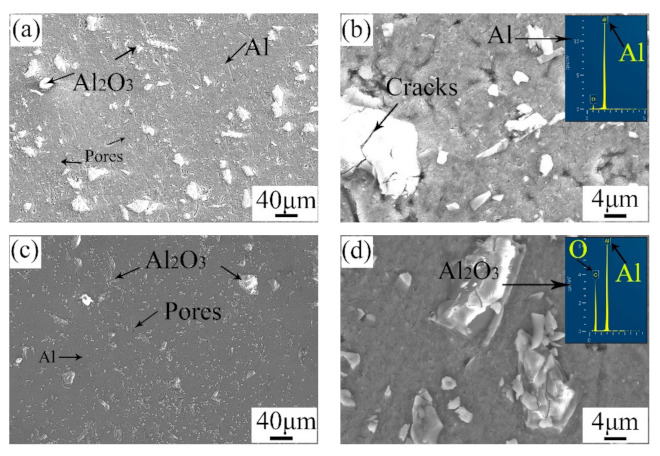
Microstructure of (**a**,**b**) the surface and (**c**,**d**) the cross-section of the repaired areas.

Figure 5 shows the microstructure of the surface and the cross-section of the repaired areas. By comparison, the microstructure of the surface was similar to that of the cross-section. A mass of angular particles was distributed in the repaired areas. According to the EDS results, the angular particles were Al_2_O_3_, and the other zones were pure Al. During the cold spray process, the mixed powders were ejected from the spray gun and hit the substrate violently. Thus, this violent impact caused a plastic deformation in the pure Al particles, as well as the crashing of the Al_2_O_3_ particles. Consequently, the pure Al particles lost their primary morphology, and many Al_2_O_3_ particles with small sizes appeared. It is mentioned that there were more tiny Al_2_O_3_ particles in the cross-section than in the surface. The outer tamping caused a great impact on the inner area. The greater the amount of tamping is, the stronger the impact on the inner area will be [24]. As a result, the Al_2_O_3_ particles in the cross-section were smaller. Meanwhile, the microstructure in the cross-section was denser than that on the surface. In addition, micro-cracks were observed around the Al_2_O_3_ particles. The porosity of the repaired areas was analyzed with Image J. The surface porosity and the cross-section porosity were 3.206 and 2.115, respectively. The results were also in accordance with the microstructure observed in Figure 5a,c. This difference can be attributed to the effect of tamping.

### 3.2. Mechanical Properties

**Figure 6:**
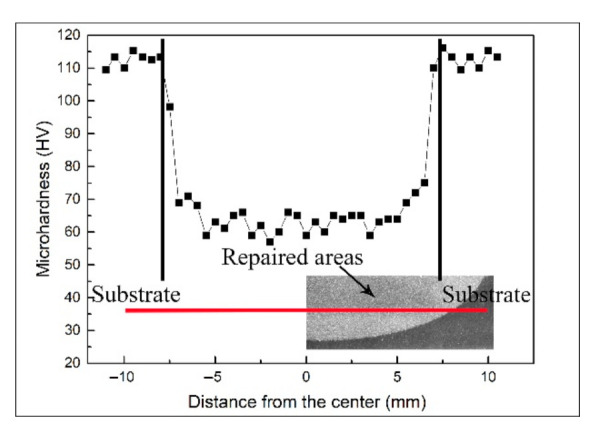
Microhardness of the samples repaired with the cold spray process.

The microhardness is an important indicator for evaluating the mechanical properties of metal. The microhardness of the samples repaired with the cold spray process is shown in Figure 6. The microhardness of the 7N01-T4 substrate was about 113.5 ± 2.2 HV, and that of the repaired areas was in the range of 57.4–63.2 HV. The variation in the microhardness was associated with the microstructure. Many Al_2_O_3_ particles existed in the repaired areas. Al_2_O_3_ particles with a high hardness can make tremendous contributions to the improvement of the microhardness. In addition, the impact of the Al_2_O_3_ particles also caused the work hardening in the deposition [25,26].

The tensile strength and elongation of samples are listed in Table 2. For convenience, the samples that were unrepaired and repaired with the cold spray process are labeled as UR and CS. It can be seen that the tensile strength of the samples was seriously reduced when there were defects. The tensile strength of the UR samples was 188.5 ± 5.5 MPa, which is only 43.3% of that of 7N01-T4. However, the tensile strength of the CS samples was about 55.4% of that of 7N01-T4. This shows that the tensile strength of the samples repaired with the cold spray process was markedly improved compared with that of the unrepaired samples.

**Figure 7:**
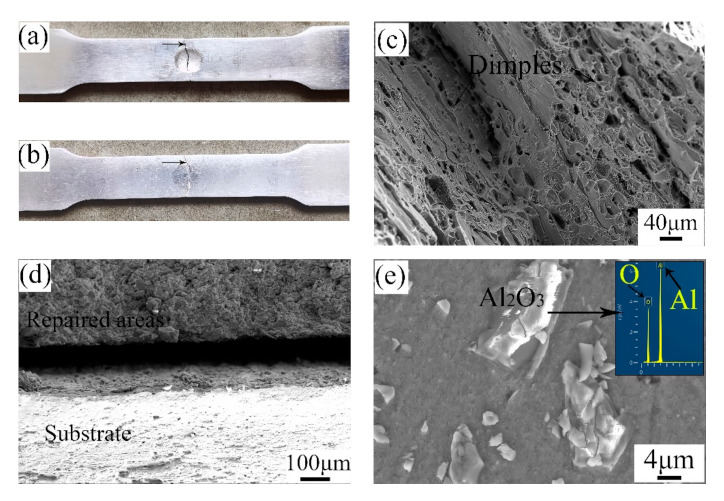
Fracture morphologies of the (**a**,**c**) UR samples and (**b**,**d**,**e**) CS samples.

Figure 7 shows the fracture morphologies of the UR samples and CS samples. As depicted, the breakages of the samples all happened in the middle of the samples. From the SEM images, it can be seen that there were numerous dimples with various sizes in the UR samples. This illustrates that the fracture mode of the UR samples was that of a ductile fracture. Likewise, the substrate of the CS samples showed a typical ductile fracture on account of the existence of many dimples. By observing the fracture of the repaired areas, the Al_2_O_3_ particles are clear and distinct. The repair process is actually a deposition process in which the raw powders are combined in the form of a mechanical bond caused by plastic deformation [27]. Therefore, the breakage first happened in the interface between pure Al and Al_2_O_3_ particles. The fracture mode of the repaired areas in the CS samples was that of a brittle fracture.

### 3.3. Corrosion Behavior

The alternate immersion tests of the samples were carried out in a salt solution for 504 h. The rates of mass loss after the tests are given in Table 3. By comparison, the UR samples had the highest rate of mass loss after the alternate immersion tests for 168 h, as well as 504 h. This can be attributed to the larger superficial area, as more corrosion could happen. In addition, the unabridged and protective corrosion layers were difficult to form during the corrosion process because of the edge between the pit and the substrate. The rate of mass loss of the CS samples was slightly lower than that of 7N01-T4. This means that the CS samples showed better corrosion resistance than the other samples.

**Figure 8:**
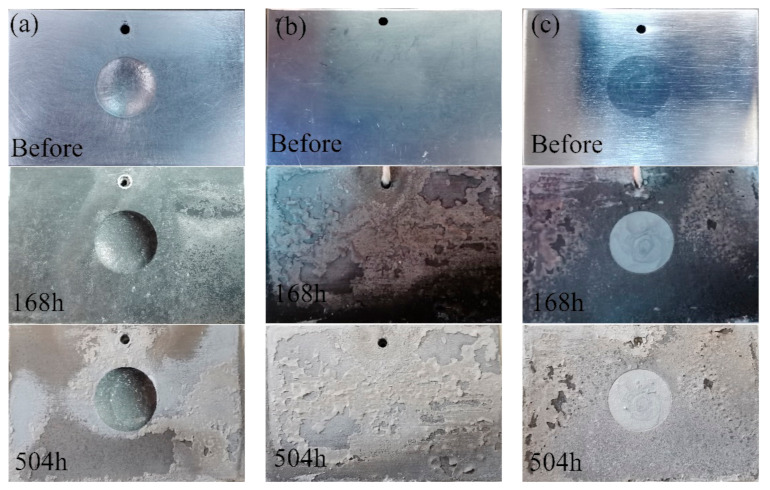
Surface appearances of (**a**) the UR samples, (**b**) 7N01-T4, and (**c**) the CS samples after alternate immersion tests.

Figure 8 shows the surface appearances of the samples after the alternate immersion tests. Clearly, all of the samples lost their metallic luster and looked dark after the alternate immersion tests for 168 h. In addition, some white corrosion products were observed in the substrate. The repaired areas in the CS samples were much brighter and showed a better surface appearance compared with the nearby substrate. After alternate immersion tests for 504 h, the 7N01-T4 samples and the CS samples were mostly covered with white products. The repaired areas in the CS samples still had brighter surfaces. This suggests that the corrosion products on the 7N01-T4 samples and the CS samples had a protective effect. When observing the UR samples, there was only a small quantity of white products in the substrate, far from the pit. The pit was in as terrible of a condition as before. Hence, there were no dense or protective corrosion layers on the surfaces of the UR samples during the corrosion process.

**Figure 9:**
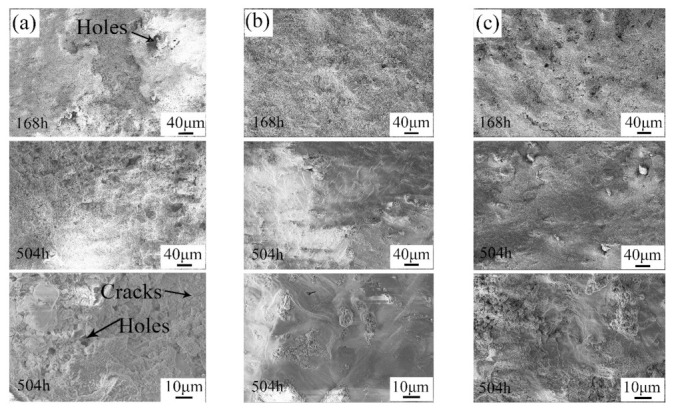
Microstructures of the corrosion layers of (**a**) the pit in the UR samples, (**b**) 7N01-T4, and (**c**) the repaired areas in the CS samples after alternate immersion tests.

The microstructures of the corrosion layers of the samples after the alternate immersion tests are shown in Figure 9. As can be seen in Figure 9a, the surface of the pit in the UR samples was covered with grainy corrosion products. Moreover, the corrosion products were loose, and many holes were clearly observed. In this case, it was easy for the corrosion medium to enter into the inner area. In contrast, the corrosion products on the surface of the 7N01-T4 samples and the CS samples were much denser. Furthermore, it was shown that the corrosion products on the surface of the 7N01-T4 samples and the CS samples linked up after alternate immersion tests for 504 h. As a result, the number of holes decreased. This structure would give tremendous protection to the fresh inner metal [28,29]. However, there were still some holes and cracks in the corrosion products in the UR samples. This also proved that the corrosion products on the surface of the UR samples still had a negative effect on the inhibition of the corrosion process. Based on the above analysis, the UR samples suffered serious corrosion in the positions where the defects were. After being repaired with the cold spray process, the corrosion resistance of the repaired samples was superior to that of 7N01-T4.

**Figure 10:**
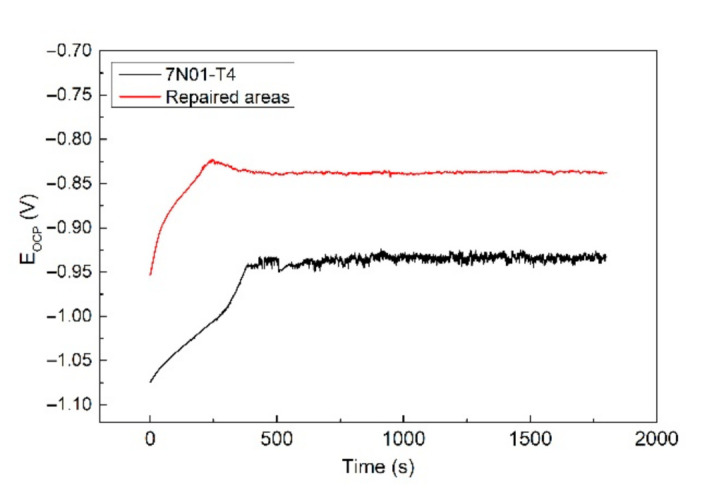
The open circuit potential of the samples before the alternate immersion tests.

Electrochemical tests were conducted in order to study the corrosion behavior of the CS samples. The CS samples for the electrochemical tests were taken entirely from the repaired areas. Figure 10 shows the open circuit potential of the samples before the alternate immersion tests. It shows that the *E*_OCP_ curves of both samples were similar. The *E*_OCP_ had a repaid increase in the initial stage and tended to be subsequently stable. The rapid increase in the *E*_OCP_ could be attributed to the passivation process on the fresh samples. Then, a passive film was formed, resulting in a stable *E*_OCP_. By comparison, the *E*_OCP_ of the repaired areas was apparently higher than that of 7N01-T4. This indicates that the repaired areas had a lower corrosion tendency in comparison to 7N01-T4 [30]. In other words, there would be a potential difference between the repaired areas and the nearby substrate in the CS samples. Thus, there remained a galvanic corrosion between the repaired areas and the nearby substrate. The repaired areas were protected as a cathode, and the nearby substrate was corroded as an anode.

**Figure 11:**
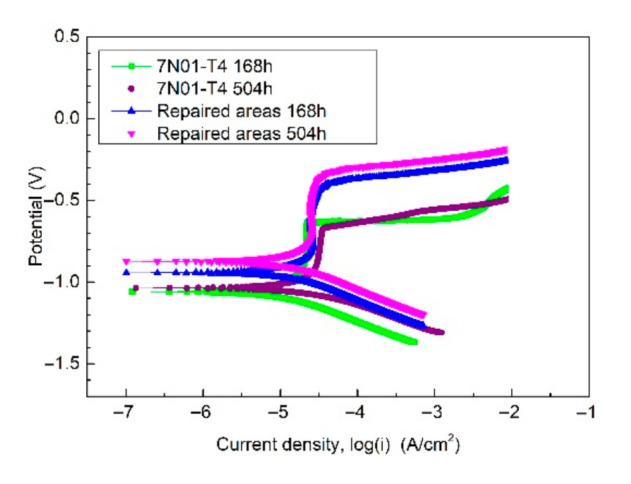
The potentiodynamic polarization curves of the samples after the alternate immersion tests.

Figure 11 shows the potentiodynamic polarization curves of the samples after the alternate immersion tests. Table 4 gives the related electrochemical parameters. As depicted, the repaired areas in the CS samples showed better corrosion resistance compared with 7N01-T4 due to the result that the *E*_corr_ was higher and the *I_c_*_orr_ was lower after the corrosion tests not only for 168 h, but also for 504 h. In addition, there was a small increase in *E*_corr_ and a small decrease in *I_c_*_orr_ after the alternate immersion tests for 504 h. So, the corrosion products on the samples had a positive effect on preventing the inner substrate from being corroded by the corrosive medium.

The CS samples in the alternate immersion tests suffered in two stages: an immersion stage and a drying stage. The immersion stage involved the process of corrosion of the samples in the salt solution, and the drying stage involved the process of corrosion of the samples in a thin liquid film. There were many ${\mathrm{HSO}}_{3}^{-}$, ${\mathrm{SO}}_{3}^{2-}$, ${\mathrm{SO}}_{4}^{2-}$, and Cl^−^ ions in the salt solution, and the pH of the solution was 3.5. Therefore, the surface oxidation film of the samples that is formed in the atmosphere was destroyed in a short time. Al is easy to dissolve and to turn into Al^3+^. The dissolution of Al usually tends to happen in positions in which there are pores and cracks [31]. The corrosion reactions in the immersion stage were as follows [32,33,34,35]:

(2) $$\mathrm{Al}\to {\mathrm{Al}}^{3+}+3e^{-}$$

(3) $${\mathrm{HSO}}_{3}^{-}\to H^{+}+{\mathrm{SO}}_{3}^{2-},$$

(4) $$2\mathrm{Al}3^{+}+3{\mathrm{SO}}_{3}^{2-}+xH_{2}O\to {\mathrm{Al}}_{2}({\mathrm{SO}}_{3}{)}_{3}\cdot xH_{2}O,$$

(5) $$2\mathrm{Al}3^{+}+3{\mathrm{SO}}_{4}^{2-}+xH_{2}O\to {\mathrm{Al}}_{2}({{\mathrm{SO}}_{4})}_{3}\cdot xH_{2}O,$$

(6) $${\mathrm{Al}}^{3+}+{\mathrm{OH}}^{-}\to \mathrm{Al}({\mathrm{OH})}_{3},$$

(7) $$O^{2}+H_{2}O+3e^{-}\to {\mathrm{4OH}}^{-}$$

Consequently, after the immersion stage, the samples were covered with Al(OH)_3_, Al_2_(SO_3_)_3_·*x*H_2_O, and Al_2_(SO_4_)_3_·*x*H_2_O. The thickness of the liquid film on the samples decreased continuously in the drying stage. The samples underwent humid atmospheric corrosion and dry atmospheric corrosion successively. Thus, more oxygen would dissolve in the liquid film. ${\mathrm{SO}}_{3}^{2-}$ ions reacted with oxygen and transformed into ${\mathrm{SO}}_{4}^{2-}$. As a result, more Al_2_(SO_4_)_3_·*x*H_2_O was formed by the reaction (5). Furthermore, the depolarization of oxygen caused by the dissolved oxygen would accelerate the dissolution of Al. At the end of the drying stage, the samples were absolutely dry, and the surface temperature went up. The corrosion rate greatly decreased due to the coverage of the corrosion products. However, the corrosion products were more prone to fracture. When the samples were immersed in the solution again, the corrosion process would go on due to the cracking and the dissolution of the corrosion products [36]. The above analysis describes the process of corrosion of the samples during the alternate immersion tests.

The CS samples showed better corrosion resistance than that of 7N01-T4, though the nearby substrate in the CS samples was sacrificed as an anode. The reasons can be summed up as follows. The repaired areas in the CS samples were relatively smaller compared with the nearby substrate, so the ability to gain electrons is limited. In addition, the CS samples were covered with dense corrosion layers, resulting in the reduction of the potential difference between the repaired areas and the nearby substrate. In conclusion, the samples repaired with the cold spray process had superior corrosion resistance to that of 7N01-T4.

## 4. Conclusions

(1) Damaged samples were successfully repaired through the cold spray process with Al and Al_2_O_3_ powders. The microstructure of the repaired areas was dense, and the porosity was relatively low. The tensile strength was markedly improved compared with that of the unrepaired damaged samples.
(2) The rate of mass loss of the samples repaired with the cold spray process was lower than that of 7N01-T4. The repaired areas had a lower corrosion tendency than that of the nearby substrate. The samples repaired with the cold spray process showed better corrosion resistance.
(3) The cold spray process is a promising method for repairing damage in aluminum alloy structures.

## Figures and Tables

**Table 1:** Chemical compositions of 7N01 (wt.%).

Si	Fe	Cu	Mn	Mg	Cr	Zn	Ti	Al
≤0.30	≤0.35	≤0.20	0.2–0.7	1.0–2.0	≤0.30	4.0–5.0	≤0.20	Bal.

**Table 2:** Tensile strength and elongation of samples.

Materials	Tensile Strength/MPa	Elongation/%
7N01-T4	435.1 ± 3.4	17.2 ± 2.3
UR	188.5 ± 5.5	3.8 ± 1.1
CS	241.4 ± 10.1	2.6 ± 1.2

**Table 3:** The rates of mass loss after the alternate immersion tests.

Materials	Rate of Mass Loss/mg·cm^−2^	
	168 h	504 h
7N01-T4	0.1279	0.2023
UR	0.2151	0.3057
CS	0.1029	0.1558

**Table 4:** Related electrochemical parameters of the samples.

Materials	*E*_corr_ (*V*_SCE_)		*I_corr_* (μA/cm^2^)	
	168 h	504 h	168 h	504 h
7N01	−1.037	−1.004	0.9301	0.8213
The repaired areas in CS samples	−0.965	−0.893	0.7004	0.6021

## Data Availability

Not applicable.
